# MH-MetroNet—A Multi-Head CNN for Passenger-Crowd Attendance Estimation

**DOI:** 10.3390/jimaging6070062

**Published:** 2020-07-02

**Authors:** Pier Luigi Mazzeo, Riccardo Contino, Paolo Spagnolo, Cosimo Distante, Ettore Stella, Massimiliano Nitti, Vito Renò

**Affiliations:** 1 Institute of Applied Sciences and Intelligent Systems, National Research Council of Italy, 73100 Lecce, Italy; pierluigi.mazzeo@cnr.it (P.L.M.); riccardo.contino@studenti.unisalento.it (R.C.); paolo.spagnolo@cnr.it (P.S.); 2 Institute of Intelligent Industrial Systems and Technologies for Advanced Manufacturing, National Research Council of Italy, Via Amendola 122 D/O, 70126 Bari, Italy; ettore.stella@cnr.it (E.S.); massimiliano.nitti@cnr.it (M.N.); vito.reno@cnr.it (V.R.)

**Keywords:** crowd counting, convolutional neural network, multi-head, smart cities, artificial intelligence

## Abstract

Knowing an accurate passengers attendance estimation on each metro car contributes to the safely coordination and sorting the crowd-passenger in each metro station. In this work we propose a multi-head Convolutional Neural Network (CNN) architecture trained to infer an estimation of passenger attendance in a metro car. The proposed network architecture consists of two main parts: a convolutional backbone, which extracts features over the whole input image, and a multi-head layers able to estimate a density map, needed to predict the number of people within the crowd image. The network performance is first evaluated on publicly available crowd counting datasets, including the ShanghaiTech part_A, ShanghaiTech part_B and UCF_CC_50, and then trained and tested on our dataset acquired in subway cars in Italy. In both cases a comparison is made against the most relevant and latest state of the art crowd counting architectures, showing that our proposed MH-MetroNet architecture outperforms in terms of Mean Absolute Error (MAE) and Mean Square Error (MSE) and passenger-crowd people number prediction.

## 1. Introduction

Smart Cities are becoming real day by day, connecting the physical world to the virtual world, and, concepts such as the crowd monitoring and managing are needful. This way crowd analysis, is becoming a hot topic in artificial intelligence because of its strong value applied in many smart cities tasks: video surveillance, public safety, urban planning, behavior understanding and so on. Real time crowd information can be usable by intelligent devices, such as smart phones and smart cameras. Ullah and his team try to detect anomalies in a flow of pedestrians in such a way as to ensure their safety [1]. Qi, Su and Aliverti in Ada-HAR make improvements to HAR (Human Activity Recognition) using smartphones by introducing an online unsupervised learning algorithm [2]. Deep Neural Network are also employed to identify tool dynamics using tool pose information in teleoperation surgery [3].

Traditional, crowd analysis methods are based on a moving window detector to detect people inside the scene [4]. Some approaches typically train a classifier by using features, such as histogram oriented gradients (HOG) [5], edges [6] and templates [7], that are extracted from the whole body. These methods behave very well in situations where people are clearly visible, but they get into difficulties in the presence of obstacles that hide parts of the person or in the presence of congested scenes. To overcome the first problem, classifiers have been developed to work on specific parts of the body, such as the head or arms [8]. These methods still do not perform well in the presence of congested scenes. Over the years, purely regressive approaches have been proposed. They try to count the number of people within the image by learning a mapping between the features extracted from individual patches of the image and the number of people within them [9]. Regression techniques used to perform the required mapping are different, among them it is possible to mention linear regression [10] and ridge regression [11]. Idrees et al, in their work [12], pointed out that the presence of congested scenes also creates problems for regressive models. This type of approach tries to obtain a mapping between the number of people and the features that are extracted, but does not take into account spatial information. For this reason, other works proposed to consider spatial information creating a density map useful to understand how people are distributed in the scene. Lempitsky and his team proposed to learn a linear mapping between features extracted from local patches and corresponding density maps [13]. Thanks to the growth of deep learning, in last years, deep neural networks have been developed to obtain both a density map and an accurate count estimation. In 2016, Zhang et al. proposed an approach based on multicolumn neural networks (**MCNN**) [14]. The concept of multicolumn neural network has been exploited by other works over the years. In particular Boominathan et al. have presented the so-called **CrowdNet** [15]. It used a reduced version of the VGG-16 [16] in which the fully connected layers have been removed. **Hydra CNN** [17] is a neural network proposed by Onoro-Rubio and Lopez-Sastre. It is defined as a scale aware counting model, it is able to estimate density maps not only of people, but also of vehicles or other objects in general. In 2017, Sam et al. proposed a new neural network for the crowd counting named **Switching CNN** [18]. It is still inspired by Reference [14], but it is composed of three different branches, each formed by five convolutional layers and 2 maxpooling layers. Sindagi and Patel proposed a cascading architecture **CMTL** (CNN-based Cascaded Multi-task Learning) [19] that estimates density map and contextually classifies crowd count in 10 different ways. In their ECCV 2018 paper, Cao et al. presented the Scale Aggregation Network (**SANET**) [20] based on multi-scale feature representation and high-resolution density maps. Zhang et al. proposed a Scale-Adaptive Convolutional Neural Network introducing a double loss, the loss on the density map and the loss on the relative count (**SaCNN**) [21]. In Reference [22], an Adversarial Cross-Scale Consistency Pursuit Network (**ACSCPNet**), is described that used the so-called adversarial loss, a loss in which both Generators G and discriminators D come into play. Li et al. proposed a network called **CSRNet** which stands for Congested Scene Recognition Network [23]. It is based on dilated filters in order to extract deeper information keeping output of the same size of the backbone output. Density Independent and Scale Aware Model (**DISAM**) proposed by Khan et al. is characterized by three main components. A first module generating proposals aware scale objects, the second identifies the heads within the scene and the third through the non-maximal suppression method tries to improve the location of the heads [24]. Tian et al. proposed **PaDNet** which stands for Pan Density Network [25]. It is characterized by a Spatial Pyramid Pooling and a fully connected layer able to extract local and global crowd density information. Sindagi and Patel improved prevoius work proposing a model called **CG-DRCN** which stands for Confidence Guided Deep Residual Crowd Counting [26]. It used part of the first five convolutional levels (C1-C5) of VGG-16 [16] followed by a new convolutional layer (C6) and a max pooling one. Wan and Chan proposed **ADMGNet** (Adaptive Density Map Generation Network) [27]. This network based on a density map refining framework is divided into two parts: (i) a counter and a (ii) a the density map refining. Liu et al. have proposed Deep Structured Scale Integration Network, **DSSINet** [28]. The network addresses the issue of scale variation of people within the crowd image using structured representation learning. Yan et al. proposed Perspective-Guided Convolution Networks **PGCNet** [29] which consists of three parts: a backbone, a perspective estimation network (PENet), and density map predictor network (DMPNet). Chen et al. presented Scale Pyramid Network (**SPN**) [30] in which proposed a single column structure composed by two blocks: a backbone and a Scale Pyramid Network. The main contribution of this work is:

- the proposed **MH-MetroNet** innovative architecture that introduction of multi-head layers for density crowd estimation
- **MH-MetroNet** outperforms the leading state of the art techniques on established publicly available datasets
- the introduction of crowd density estimation in subway cars where the best performing architecture is **MH-MetroNet** which outperforms the latest state-of-the-art architectures where publicly code is available.

The remainder of this paper is organized as follows. Section 1 contains a review of the state of the art. Section 2 contains the mathematical problem formulation. Section 3 presents the proposed methodology. Section 4 shows extensive experimental results. Section 5 reports conclusions.

## 2. Problem Formulation

Given a set *N* of training crowd images $I_{i}$ we generate a ground-truth density map $D_{i}^{gt}$. *N* depends on the number of images in the dataset that is used. In this work it varies from a minimum of 50 for the UCF_CC_50 dataset to a maximum of 2823 in the case of the private dataset.

For each image is annotated the number of people involved $s_{i}$ and the position of the head of each of them $P_{i}^{gt}$ = {$p_{1}$, $p_{2}$, …, $p_{c_{i}}$}. For the public datasets these positions are already provided, for the private dataset it was necessary to annotate them using the YOLOv3 neural network. Ground-truth density map from it is generated as proposed in Reference [23]. A head to the $x_{j}$ pixel, it is represented as a delta function δ(*x* − $x_{j}$ ). An image with $c_{i}$ heads labeled can be represented as a function:

(1) $$H\left(x\right)=\sum_{i=1}^{c_{i}}\delta \left(x-x_{j}\right).$$

In this way a matrix is obtained with peaks where there is a head. In order to spread this peak in a smoothed density function we perform a convolution with a Gaussian $G_{\sigma }$ kernel:

(2) $$D_{i}^{gt}\left(x\right)=H\left(x\right)*G\left(x\right).$$

Thus the peak is distributed over the neighboring pixels.

This way we assume that each pixel is an independent sample in the image plane that does not represent the real case. In order to avoid this, for each head in the image we calculate the distance to its nearest neighbors k and the average distance $\bar{d}$ is derived. At this point we use a Gaussian filter with σ proportional to the average distance $\bar{d}$:

(3) $$D_{i}^{gt}\left(x\right)=H\left(x\right)*G_{\sigma }\left(x\right).$$

The result of these operations is saved in a csv or h5py file to be easily used during network training.

### Training Strategy

The goal is to learn a non-linear mapping *F* that, given in input a crowd image, provides in output an estimated density map $D_{i}^{est}$ that is as close as possible to the ground truth $D_{i}^{gt}$ in terms of $L^{2}$ norm:

(4) $$L\left(\theta \right)=\frac{1}{2N}\sum_{i=1}^{N}{∥F\left(X_{i},\theta \right)-D_{i}^{gt}\left(X_{i}\right)∥}_{2},$$

where $X_{i}$ is the *i*th training sample, θ is a set of network parameters (weights and biases) and $F(X_{i},\theta )$ is the estimated density map.

Once the density map has been obtained, it is possible to make an estimate of the count by performing an integration (sum). Training and evaluation was performed on NVIDIA RTX TITAN GPU using the PyTorch framework. In the training phase Adam optimization was used with a learning rate that is decreased at each epoch and an initial value equal to 0.00001.

## 3. Network Architecture

**Figure 1:**
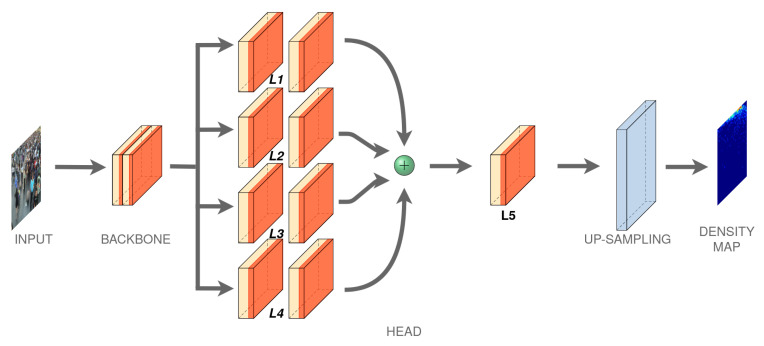
Proposed **MH-MetroNet** architecture.

We estimate this density map by designing a convolutional neural network composed of: (i) a backbone for features extraction and (ii) a multi-head layers that combining these features obtaining a density map which contains an estimation of people number of the input crowd images. A scheme of the proposed architecture is showed in Figure 1.

### 3.1. Backbone

Over the years, increasingly deep and complex networks have been developed to improve their performance. Usually a pre-trained model is used and its performance is improved by performing a fine-tuning operation or new levels or improved techniques are used to make the network deeper. Many of the works seen in Section 1 use the VGG-16 network as a backbone, in this paper different backbones have been used to try to achieve better performance both in terms of density map estimation than in terms of counting.

#### 3.1.1. DenseNet

DenseNet is a network proposed by Huang and his team [31]. The model used in this work is a restricted version of the DenseNet-121. In particular, only the first levels are used, that is, those up to the Dens 2 block. This is because the network has been developed to solve classification problems and the size of the image tends to decrease with each layer. The version used reduces the size by one eighth compared to the original ones.

#### 3.1.2. ResNet

The ResNet is a family of architecture proposed in Reference [32] based on the VGG network where the convolutional layers have mostly a 3×3 kernel size and uses the so-called Residual Learning to improve performance and results. The ResNet family consists of five different configurations, from the 18-layer version to the 152-layer version. As the depth of the network increases, performance improves, but training slows down.

The version used in this work is ResNet-152. In particular, the layers are removed from the fourth onward. In this way the backbone provides an image in output with dimensions equal to one eighth to the original ones.

#### 3.1.3. SeNet

SeNet, presented by Hu and his team in Reference [33], introduces a new block called Squeeze And Excitation, useful to improve channel interdependence with almost no computational cost enhancing the performance. The block takes in input the tensor, performs an average pooling, a fully connected level is applied followed by a ReLU, finally a further fully connected level followed by a sigmoid. The output of this block is scaled to be joined to the input tensor. It is possible to construct an SE network simply by stacking a collection of SE blocks one after another.

A reduced version of the Senet-154 is used in this work. In particular, all layers starting from layer 3 are deleted. In this way the backbone is much lighter and allows to obtain an image of one eighth the original size.

#### 3.1.4. EfficientNet

Mingxing Tan and Quoc V. LE proposed a new architecture in 2019 called EfficientNet [34]. This new network is able to achieve better performance in the ImageNet dataset classification problem while requiring less computational effort. The authors have created a family of EfficientNet starting from the B0 architecture up to the B7 by tuning in a suitable way the network depth, width and resolution. The model used in this work is a restricted version of the EfficientNet-B5. In particular, only the first levels are used, that is, those up to MBConvBlock 9.Like the other backbones also in this case a reduced version is used in order to obtain an output image with dimensions equal to one eighth of the original ones.

### 3.2. Head Layers

The features extracted from the backbone are used to obtain a density map representation by passing through a four parallel blocks ($L_{1},\ldots ,L_{4}$). Each block contains two convolutional layers with different filter kernel-sizes. In the block, the first layer has filter of 3×3 or 5×5 with different channels, while the following convolutional layer of the $L_{i}$ block compress the depths at the same values, that is, 32 channels. (Table 1) shows the four parallel block configurations, where it is shown that information along channels in the second layer is squeezed to a depth of 32 and merged using an average or a concatenation (concatenation produces slightly better results). An additional convolution layer $L_{5}$ is used to obtain a single activation map (resulted form a conv1-1 operation) which represents a first subsampled estimation of the density map. This has a spatial resolution equal to one eighth of the original image size. To obtain a higher resolution map, an UpSampling is performed by an interpolation operation. The interpolation operation gives better results towards the usual unpooling operations or dilated convolutions.

## 4. Experimental Results

We demonstrate the proposed architecture in three sub-experiments the first on three public available datasets, UCF_CC_50 [12], ShanghaiTech part_A, ShanghaiTech part_B [14] changing different backbones, the second on the same datasets against the previous state of the art method, the third evaluating our approach on the novel subway cars dataset. The MH-MetroNet network architecture used for the training phase is that described in Section 3. In particular, different well-known backbones have been used—ResNet-152 [32], DenseNet-121 [31], SeNet-154 [33], EfficientNet-b5 [34].

### 4.1. Datasets

In the field of crowd analysis some public datasets have been proposed to evaluate the performance of the developed models. Three of them have been chosen to train and evaluate the performance of the proposed network, in particular ShanghaiTech_partA, ShanghaiTech_partB and UCF_CC_50. In addition, a private dataset containing images of subway cars acquired in Naples (Italy) has been used.

### 4.2. UCF_CC_50 Dataset

**Figure 2:**
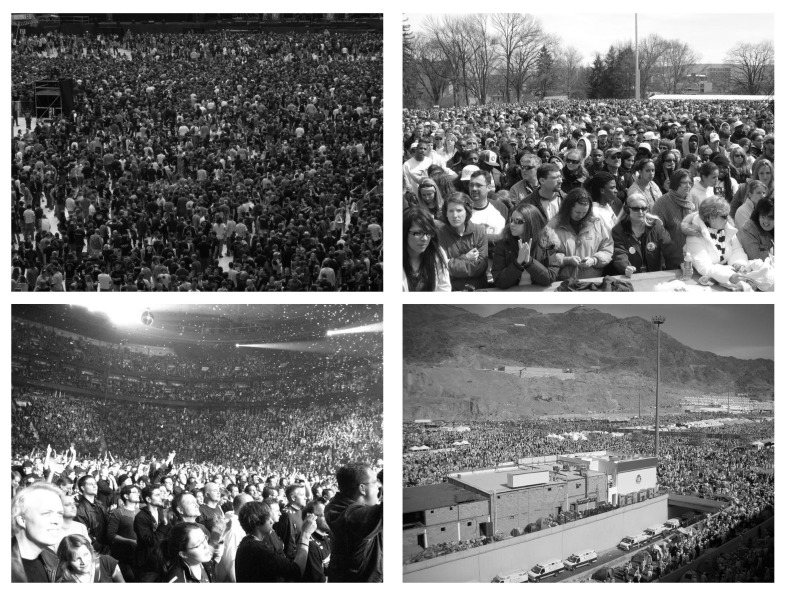
UCF_CC_50 crowd images [12].

UCF_CC_50 is a dataset provided by Idrees et al. [12]. It contains 50 black and white images of very congested scenes of different resolution (from 368×496 to 1024×1024) extracted from the web, mostly from FLICKR. The dataset contains a total of 63.974 individuals, averaging 1.279 per image. Some of the images in the dataset are shown in Figure 2.

### 4.3. ShanghaiTech Dataset

The ShanghaiTech dataset has been introduced by Zhang et al. in 2016 [14]. It is divided into two parts: part A and part B, for a total of 1198 images of congested crowd noted and 330,165 people.

#### 4.3.1. ShanghaiTech Part A Dataset

The ShanghaiTech part A dataset consists of a total of 482 images with different resolutions (from 300×200 to 1024×1024 pixel), 300 of which are train images and 182 test images. The number of people within the dataset varies greatly from a few tens to a few thousand.

**Figure 3:**
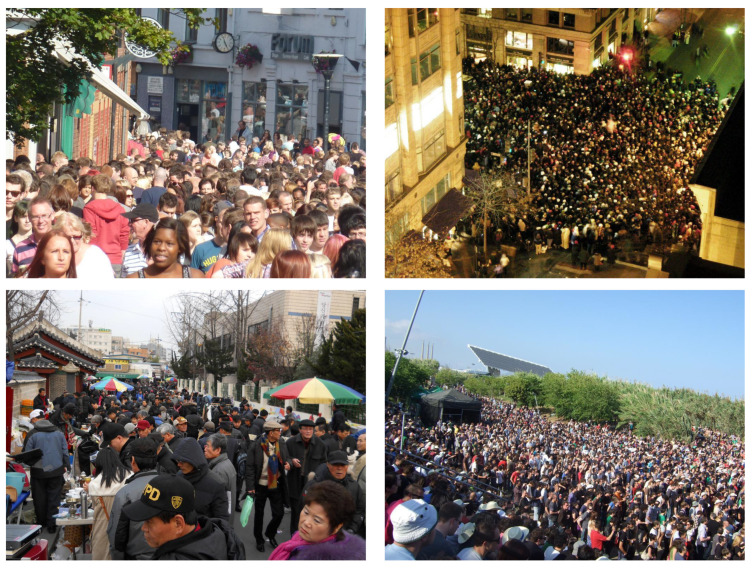
ShanghaiTech part A crowd images [14].

Some of the images in the dataset are shown in Figure 3.

#### 4.3.2. ShanghaiTech Part B Dataset

The ShanghaiTech part B dataset consists of a total of 716 images with the same resolutions (1024×768 pixel), 400 of which are train images and 316 test images. In this case the number of people is much lower than in Part A, in fact it varies from a few units up to a few hundreds.

**Figure 4:**
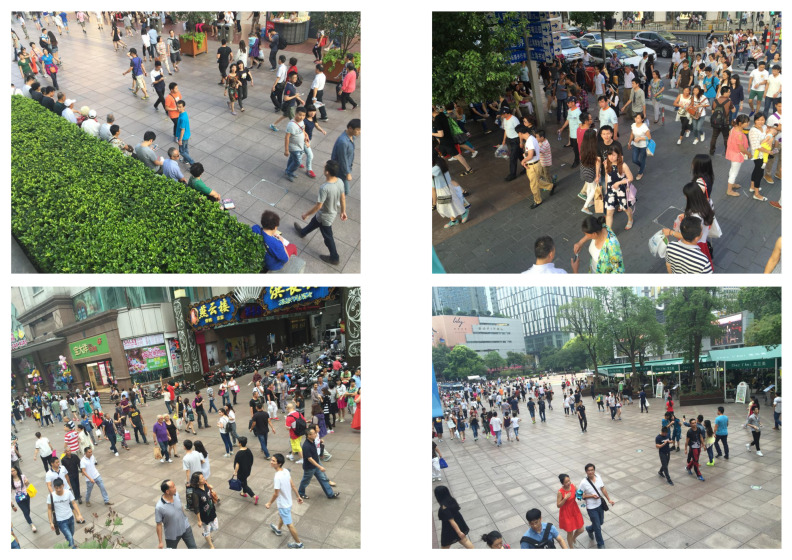
ShanghaiTech part B crowd images [14].

Some of the images in the dataset are shown in Figure 4.

### 4.4. Subway Cars Dataset

**Figure 5:**
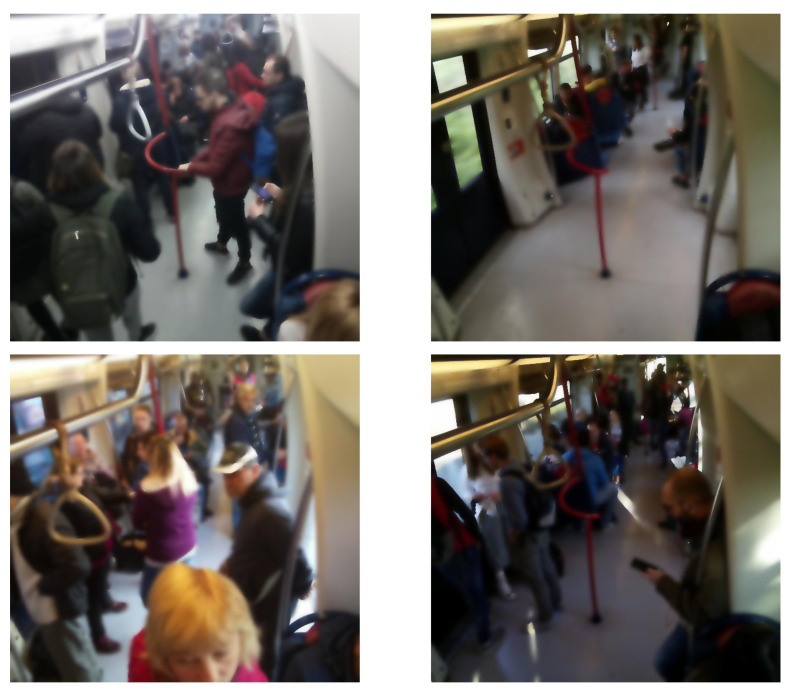
Subway cars passenger-crowd images.

The dataset proposed in this work contains images acquired from metropolitan wagons in Naples (Italy). The dataset is composed of a total of 2823 images of the same resolution (640×600 pixel), 1886 of which are train images, 474 validation images and 463 test images, involving 16,070 people. The dataset is unbalanced, in particular, cases where the wagon is full are less than in a partially full car situation. There are also images where the wagon is empty, this represents a real challenge for the neural networks, as they are not designed to analyze empty spaces. Sample images of this dataset are shown in Figure 5.

### 4.5. Metrics

In this section the two evaluation techniques of the results obtained are analyzed: **MAE** (*Mean Absolute Error*) and **MSE** (*Mean Squared Error*).

#### 4.5.1. Mean Absolute Error

The MAE, also known as L1 loss, is an average of the error that is made on the estimate of the people count, that is, the difference between the calculated value and the real value. Formally it is defined as follows:

(5) $$MAE\;=\;\frac{1}{N}\;\sum_{i=1}^{N}\;\left|c_{i}^{est}-c_{i}^{gt}\right|,$$

where N is the number of test samples, $c_{i}^{est}$ is estimated count and $c_{i}^{gt}$ is ground-truth count corresponding to the *i*th sample.

The advantage of this metric is its easy interpretation and its high resistance to outliers.

#### 4.5.2. Mean Squared Error

The MSE, also known as L2 loss, is an average of the squared error that is made on the estimate of the people count, that is, the difference between the calculated value and the real value. Formally it is defined as follows:

(6) $$MSE\;=\;\frac{1}{N}\;\sum_{i=1}^{N}\;{\left(c_{i}^{est}-c_{i}^{gt}\right)}^{2},$$

where N is the number of test samples, $c_{i}^{est}$ is estimated count and $c_{i}^{gt}$ is ground-truth count corresponding to the *i*th sample.

This metric is very sensitive to large errors. For this reason it is useful to assess whether the model is wrong a lot in specific circumstances.

### 4.6. Results on UCF_CC_50 Dataset

**Figure 6:**
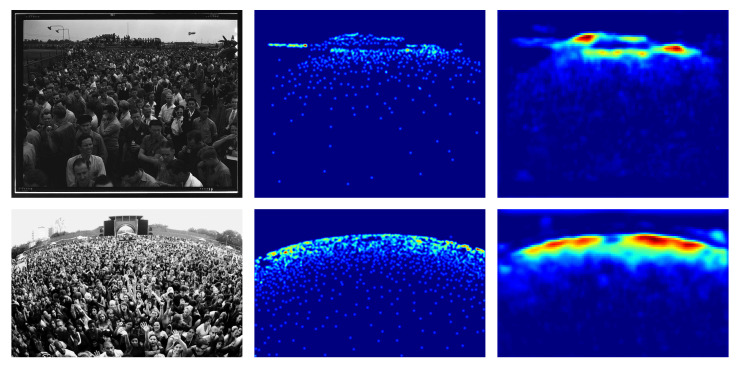
UCF_CC_50 density map estimation examples, (**Left**) the crowd image, (**Center**) ground-truth density map, (**Right**) estimated density map.

The UCF_CC_50 dataset is extremely challenging due to the few images proposed. In Table 2 it can be seen the performance achieved using different backbones. ResNet-152 backbone obtains the best results both in terms of MAE and MSE. Examples of the density map produced on this dataset are shown in Figure 6.

### 4.7. Results on ShanghaiTech Part A and B Datasets

**Figure 7:**
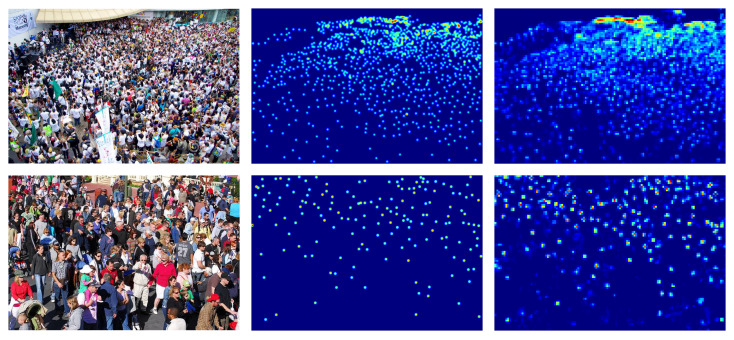
ShanghaiTech part A density map estimation examples. Left the crowd image, middle ground-truth density map, right estimated density map.

**Figure 8:**
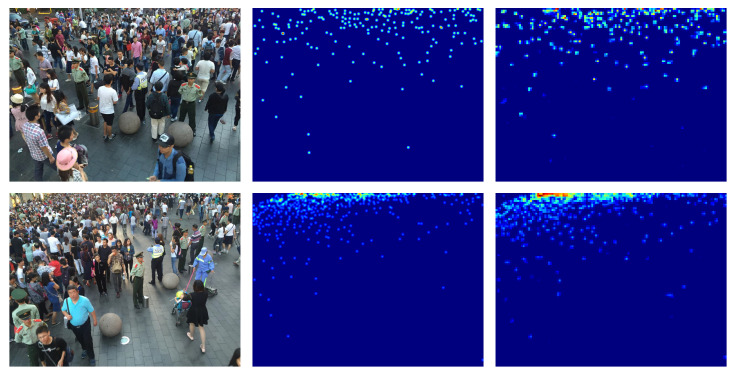
ShanghaiTech part B density map estimation examples. Left the crowd image, middle ground-truth density map, right estimated density map.

The ShanghaiTech part_A dataset contains images of different resolutions with a high number of people. The ShanghaiTech part_B dataset contains images of the same resolution with much fewer people than ShanghaiTech part A. In Table 3 it is possible to see the performance reached by the network using different backbones. Also on these datasets our architecture with ResNet-152 backbone achieves the best results in terms of MAE and MSE. Examples of the density map produced on this dataset are shown in Figure 7 and Figure 8.

### 4.8. Results Comparison

The results contained in Table 2 and Table 3 clearly demonstrate that the proposed architecture with resnet-152 backbone achieves the best performance in terms of MAE and MSE. Table 4 and Table 5 show how the proposed architecture achieves promising results compared to the state of the art methodologies, in fact we obtain the best result for the UCF_CC_50 dataset (Table 4) and the one closest to the best for the ShanghaiTech datasets (Table 5).

**Figure 9:**
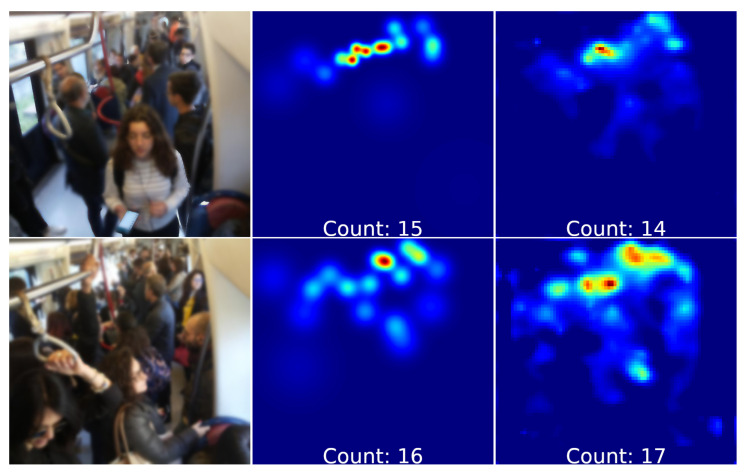
Subway cars dataset density map estimation examples:(**Left**) the crowd image, (**center**) ground-truth density map, (**right**) estimated density map (with passengers count).

This dataset contains images of the inside metropolitan wagons where are a smaller number of people, compared to the other datasets. Table 6 describes a performance comparison among a subset of state of art work (i.e., only ones with code source public available) and the proposed architecture on this novel dataset. It can be noted that removing the upsampling block we obtain a density map of reduced size, equal to one eighth of the original and slightly better results in terms of MAE and MSE. Anyway our architecture gives the best results on Subway cars dataset. A density map generated by our network architecture of crowd passengers is showed in Figure 9.

## 5. Conclusions

This paper proposed a new a multi-head CNN architecture able to perform crowd analysis estimating the density map and counting the number of people. We used as head layers four parallel convolutional blocks able to aggregate features from the conv-backbone in order to obtain a high fidelity density map estimation. We demonstrated our model in two crowd counting datasets and outperformed in terms of performance the leading latest state-of-the-art architectures. We also extended our model to crowd-passengers counting task and our model achieved the best accuracy as well. Source code is available to https://bitbucket.org/isasi-lecce/mh-metronet/src/master/.

Future work will be focused on a new module called Attention Block, based on the Attention mechanism. This module, first used in natural language processing, is also being adopted in computer vision and promises to improve performance by lowering complexity.

## Figures and Tables

**Table 1:** Configuration of the Multi-Head layers of the proposed **MH-MetroNet** architecture (Figure 1). Each block is composed of two convolutional layers except for $L_{5}$ (only one conv layer) and their parameters are denoted as conv-(kernel size)-(number of filters).

Multi-Head Blocks Config		
$L_{1}$	conv3-256	conv3-32
$L_{2}$	conv3-64	conv3-32
$L_{3}$	conv5-256	conv5-32
$L_{4}$	conv5-64	conv5-32
$L_{5}$	conv1-1	

**Table 2:** Proposed **MH-MetroNet** architecture performance with different backbones on UCF_CC_50 datasets.

Backbone	MAE	MSE
DenseNet-121 [31]	187.74	270.31
**ResNet-152** [32]	**170.0**	**221.95**
SeNet-154 [33]	181.24	257.21
EfficientNet-b5 [34]	277.48	314.73

**Table 3:** Proposed **MH-MetroNet** architecture performance with different backbones on ShanghaiTech part_A and part_B.

	Part A		Part B	
**Backbone**	**MAE**	**MSE**	**MAE**	**MSE**
DenseNet-121 [31]	82.62	133.75	8.66	13.85
**ResNet-152** [32]	**67.52**	**113.47**	**7.93**	**13.0**
SeNet-154 [33]	181.24	257.21	9.46	14.57
EfficientNet-b5 [34]	115.62	179.53	14.39	23.96

**Table 4:** UCF_CC_50 performance comparison.

Method	MAE	MSE
MCNN [14]	377.6	509.1
CrowdNet [15]	452.5	-
Hydra CNN [17]	333.73	425.26
Switching CNN [18]	318.1	439.2
CMTL [19]	322.8	397.9
SANet [20]	258.4	334.9
SaCNN [21]	314.9	424.8
ACSCP [22]	291.0	404.6
CSRNet [23]	266.1	397.5
**PADNet** [25]	**185.8**	**278.3**
DSSINet [28]	216.9	302.4
SPN [30]	259.2	335.9
**Our**	**170.0**	**221.95**

**Table 5:** ShanghaiTech part A and part B performance comparison.

Method	Part A		Part B	
	MAE	MSE	MAE	MSE
MCNN [14]	110.2	173.2	26.4	41.3
Switching CNN [18]	90.4	135.0	21.6	33.4
CMTL [19]	101.3	152.4	20.0	31.1
SANet [20]	67.0	104.5	8.4	13.6
SaCNN [21]	86.8	139.2	16.2	25.8
ACSCP [22]	75.7	102.7	17.2	27.4
CSRNet [23]	68.2	115.0	10.6	16.0
PADNet [25]	59.2	98.1	8.1	12.2
**DSSINet** [28]	60.63	96.04	**6.85**	**10.34**
**PGCNet** [29]	**57.0**	**86.0**	-	-
SPN [30]	61.7	99.5	9.4	14.4
**Our**	**67.52**	**113.47**	**7.93**	**13.0**

**Table 6:** Subway cars dataset performance comparison.

Method	MAE	MSE
MCNN [14]	3.89	4.92
CMTL [19]	4.17	5.28
CSRNet [23]	1.22	1.57
SANet [20]	0.9	1.3
**Our**	**1.12**	**1.43**
**Our no up**	**0.82**	**1.28**
